# Effect of a single intra-articular administration of stanozolol in a naturally occurring canine osteoarthritis model: a randomised trial

**DOI:** 10.1038/s41598-022-09934-y

**Published:** 2022-04-07

**Authors:** J. C. Alves, A. Santos, P. Jorge, C. Lavrador, L. Miguel Carreira

**Affiliations:** 1 Divisão de Medicina Veterinária, Guarda Nacional Republicana (GNR), Rua Presidente Arriaga, 9, 1200-771 Lisbon, Portugal; 2 MED – Mediterranean Institute for Agriculture, Environment and Development, Instituto de Investigação e Formação Avançada, Universidade de Évora, Pólo da Mitra, Ap. 94, 7006-554 Évora, Portugal; 3 Faculty of Veterinary Medicine, University of Lisbon (FMV/ULisboa), Lisbon, Portugal; 4 Interdisciplinary Centre for Research in Animal Health (CIISA), University of Lisbon (FMV/ULisboa), Lisbon, Portugal; 5 Anjos of Assis Veterinary Medicine Centre (CMVAA), Barreiro, Portugal

**Keywords:** Musculoskeletal system, Osteoarthritis

## Abstract

Osteoarthritis (OA) is a disease with a high negative impact on patient’s quality of life and a high financial burden. It is a source of chronic pain and affects all mammals, including humans and dogs. As the dog is a common model for translation research of human OA, and exploring spontaneous dog OA can improve the health and well-being of both humans and dogs. To describe the effect of the intra-articular administration of stanozolol in a naturally occurring canine OA model, forty canine (N = 40) hip joints were randomly assigned to receive stanozolol or saline (control). On treatment day and at 8, 15, 30, 90, and 180 days post-treatment, several evaluations were conducted: weight distribution, joint range of motion, thigh girth, digital thermography, and radiographic signs. Also, synovial fluid C-reactive protein and interleukin-1 levels were evaluated. Results from four Clinical Metrology Instruments was also gathered. Results were compared with Repeated Measures ANOVA, with a Huynh–Feldt correction, paired-samples t-test, or Wilcoxon signed-rank test, with *p* < 0.05. OA was graded as mild (90%), moderate (5%), and severe (5%), including both sexes. They had a mean age of 6.5 ± 2.4 years and a bodyweight of 26.7 ± 5.2 kg. No differences were found between groups at treatment day in all considered evaluations. Weight distribution showed significant improvements with stanozolol from 15 days (*p* < 0.05) up to 180 days (*p* < 0.01). Lower values during thermographic evaluation in both views taken and improved joint extension at 90 (*p* = 0.02) and 180 days (*p* < 0.01) were observed. Pain and function scores improved up to 180 days. In the control group, radiographic signs progressed, in contrast with stanozolol. The use of stanozolol was safe and produced significant improvements in weight-bearing, pain score, and clinical evaluations in a naturally occurring canine OA model.

## Introduction

Osteoarthritis (OA) is a disease spanning all species of mammals. It is particularly important in humans and dogs, being a source of chronic pain and posing a significant burden to societies. Since it has such a significant toll on the quality of life, it implies a considerable cost in healthcare. Since life expectancy and obesity of populations is increasing, the prevalence of the disease is also expected to rise^1–4^. The dog shows a similar pathologic process, clinical presentation, and response to treatment to those in humans, where degenerative, trauma, and overuse aetiologies occur, making dogs a frequent animal model for the study of OA^5^. The naturally occurring canine model, in particular, provides substantial benefits in comparison to other models. It presents a foreshortened lifespan while maintaining the same life stages of human disease, and sharing many environmental conditions with humans, specifically those that influence human OA. For those reasons, the naturally occurring canine model is easier to study^5–12^. The study of canine OA can provide important insight into the disease in a translational approach under the One Medicine initiative and improve the health and well-being of humans and dogs^11,13^.

OA is still an incurable condition, and the medical approach to its treatment aims at slowing disease progression while relieving symptoms, particularly pain, but treatment options are still limited^11,14–16^. Stanozolol is a synthetic derivative of testosterone, with properties that include anabolic/androgenic activity, probably associated with its affinity for androgenic and, at lower doses, glucocorticoid receptors^17^. It has a high androgenic potential, but its long-term use has not induced activity and aggressivity changes in mice^18^. An anti-catabolic effect potentiates stanozolol's anabolic effect at the glucocorticoide receptor level, where it behaves as a competitive antagonist of the catabolic corticosteroids^19^. In vitro human studies and ovine and equine models have described that stanozolol was able to induce fibroblasts, to increase collagen production in a dose-dependent pathway through transforming growth factor-1β synthesis while decreasing nitric oxide production and stimulating the autocrine secretion of insulin-like growth factor-1, which induces osteoblast proliferation and collagen synthesis^20–23^. In humans, an increase of transforming growth factor-1β synthesis is related to a decrease in articular pain^24^. It also demonstrated chondroprotective effects through the downregulation of genes for pro-inflammatory/catabolic cytokines and enzymes associated with OA in equine in vitro chondrocytes^25^. In an ovine surgical model of OA, intra-articular stanozolol was able to preserve the stifle joint's gross anatomy, reducing osteophyte formation, subchondral bone reaction, and promoting articular cartilage regeneration, at 3 and 9 months post-surgery^21^. In dogs, a 0.3 mg/kg dose has been described for intra-articular administration, in the management of knee OA, and oral use to treat tracheal collapse^26,27^. Before evaluating multiple administrations, as described in other animal models^21,23^, the assessment of a single administration of stanozolol is required to determine treatment safety its effect following intra-articular administration.

This study aims to compare the effect of stanozolol to a control group in a naturally occurring canine OA model. We hypothesize that stanozolol is able to reduce pain levels improve function in OA joints compared to a control group.

## Results

The sample included 40 joints of both intact males (n = 22, in 12 CG and 10 in SG) and females (n = 18, in 8 CG and 10 in SG) Police working dogs. They had with a mean age of 6.5 ± 2.4 years and bodyweight of 26.7 ± 5.2 kg. Dogs were of breeds commonly employed in police forces, similarly distributed between CG and SG: German Shepherd Dogs (n = 12, 6 in CG and 6 in SG), Labrador Retriever (n = 12, 6 in CG and 6 in SG), Belgian Malinois Shepherd Dogs (n = 10, 6 in CG and 4 in SG), and Dutch Shepherd Dog (n = 6, 4 in CG and 2 in SG). At the initial evaluation, OA was classified as mild in 36 joints (90%, in 18 CG and 18 in SG), moderate as 2 (5%, all in CG), and severe as 2 (5%, all in SG), according to the Orthopedic Foundation for Animals hip grading scheme^28^. Levene's test for homogeneity was used to control baseline values, and no differences were found between groups at the initial evaluation. All patients were evaluated in all assessment moments. Increased lameness was observed in four cases of the stanozolol group following administration, which spontaneously resolved within a few days.

**Figure 1:**
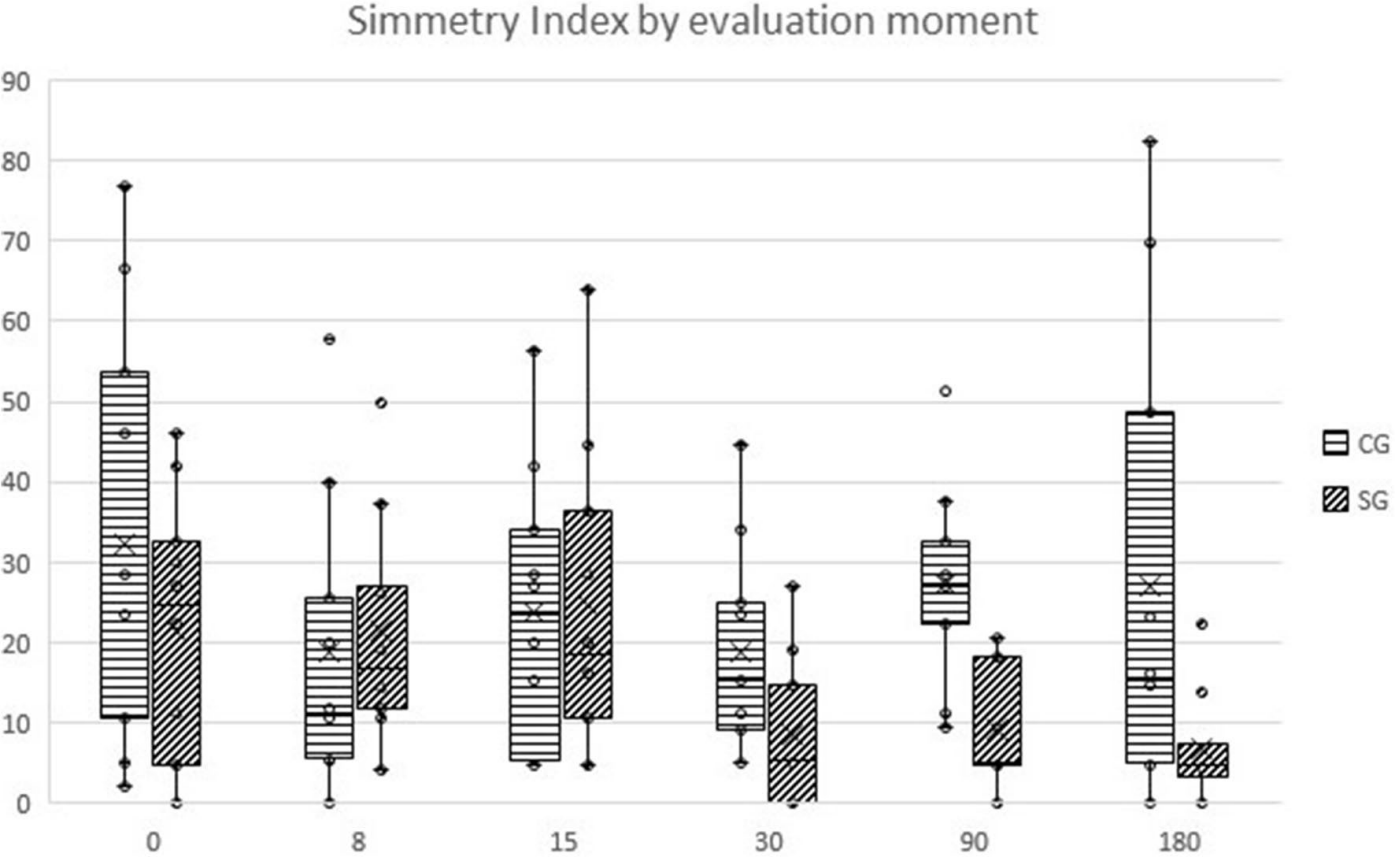
Overall evolution of Symmetry Index in the control group and treatment group. Box plots represent the median, 25th and 75th percentiles, and whiskers represent 10th and 90th percentiles.

Values recorded in stanozolol and control groups for different evaluations made throughout the study, are presented in Tables 1 and 2. Comparing results between groups with repeated measures ANOVA with a Huynh–Feldt correction, significant differences between groups were found concerning deviation (F(4.4, 140.1) = 11.2, *p* < 0.01), SI (F(3.8, 121.5) = 6.2, *p* < 0.01), mean temperature on a dorsoventral (DV) view (F(3.8, 107.8) = 4.6, *p* = 0.002), maximal temperature on a DV view (F(3.4, 95.1) = 3.7, *p* = 0.011), mean temperature on a lateral (Lt) view (F(5, 150) = 37.1, *p* < 0.001), maximal temperature on a Lt view (F(3.9, 118.2) = 123.7, *p* < 0.001), thigh girth (F(5, 170) = 6.7, *p* < 0.001) joint extension (F(3.6, 107.5) = 171.3, *p* < 0.001), joint flexion (F(5, 170) = 15.9, *p* < 0.001) and IL-1 synovial concentration (F(1.8, 64.5) = 7.4, *p* = 0.002). Evolution of SI is presented in Fig. 1.

**Table 1:** Mean values (± standard deviation) of goniometry, thigh girth evaluation, pedometer and Clinical metrology instruments evaluated throughout the study.

Parameters	Treatment day				8 days					15 days					
	Control		Stanozolol		Control		Stanozolol		*p*	Control		Stanozolol		*p*	
	Mean	SD	Mean	SD	Mean	SD	Mean	SD		Mean	SD	Mean	SD		
**Goniometry**															
Flexion (°, mean ± SD)	55,0	4.4	56.6	3.7	55.3	3.7	55.9	4.5	1.00	57.2	5.2	56.2	3.2	1.00	
Extension (°, mean ± SD)	151.2	3.9	156.9	6.0	149.9	4.6	156.9	6.0	< 0.01	151.1	3.5	115.1	6.4	1.00	
Thigh girth (cm, mean ± SD)	31.2	2.6	29.1	1.9	31.1	3.3	29.1	2.1	1.00	31.1	2.9	29.8	2.0	1.00	
Pedometer (daily steps ± SD)	1445.7	755.7	910.9	811.2	829.5	931.3	1165.2	684.5	1.00	606.0	309.5	1043.2	733.1	0.43	
**CMI**															
HVAS (0–10)	6.8	1.2	6.7	1.3	6.7	1.5	6.6	1.4	1.00	6.8	1.2	7.1	0.8	1.00	
CBPI—PSS (0–10)	3.1	1.9	2.9	1.5	3.4	2.3	3.1	2.3	0.53	3.7	2.8	1.9	2.1	0.04*	
CBPI—PIS (0–10)	3.2	2.2	2.3	1.7	3.4	2.1	2.9	1.9	0.02*	3.6	2.1	1.9	1.2	0.01*	
COI—Stiffness (0–16)	3.4	3.4	4.0	2.8	4.1	3.3	2.3	2.3	0.56	4.1	3.2	1.5	1.9	0.02*	
COI—Function (0–16)	3.6	4.1	4.0	3.6	4.1	4.0	1.8	2.1	< 0.01*	4.4	5.5	0.9	1.4	< 0.01*	
COI—Gait (0–20)	4.7	5.2	5.2	3.9	5.4	6.1	3.1	3.4	1.00	5.8	4.3	1.8	2.9	0.02*	
COI—QOL (0–12)	4.5	2.6	4.3	2.5	4.6	2.7	4.0	2.2	1.00	4.7	2.9	3.3	2.3	1.00	
COI—Overall score (0–64)	16.4	14.7	17.5	12.4	18.2	13.8	11.2	9.0	0.7	18.6	13.8	7.5	7.4	0.29	
LOAD (0–52)	13.6	10.5	8.2	5.2	14.4	12.7	11.1	7.2	0.17	14.3	10.7	11.1	7.2	0.02*	

Parameters	30 days					90 days					180 days				
	Control		Stanozolol		*p*	Control		Stanozolol		*p*	Control		Stanozolol		*p*
	Mean	SD	Mean	SD		Mean	SD	Mean	SD		Mean	SD	Mean	SD	
**Goniometry**															
Flexion (°, mean ± SD)	53.6	2.9	55.4	5.3	1.00	52.7	2.9	55.8	5.5	0.02*	51.6	2.2	50.9	1.8	< 0.01*
Extension (°, mean ± SD)	150.8	3.4	153.2	4.6	0.07	150.8	2.9	154.8	2.9	0.59	151.3	2.9	155.0	3.0	0.04*
Thigh girth (cm, mean ± SD)	30.6	2.7	28.9	1.4	1.00	31.6	2.7	32.5	2.7	1.00	31.5	2.2	29.8	1.9	1.00
Pedometer (daily steps ± SD)	594.5	663.4	869.2	1091.5	0.58	451.9	463.0	440.0	455.3	0.36	434.9	455.8	588.33	788.3	0.14
**CMI**															
HVAS (0–10)	6.4	1.4	7.1	1.3	1.00	6.6	1.7	6.6	1.3	1.00	6.5	1.4	6.9	1.2	1.00
CBPI—PSS (0–10)	3.7	2.6	2.4	1.9	< 0.05*	4.1	2.9	2.9	1.9	0.04*	3.6	3.1	2.6	1.8	0.02*
CBPI—PIS (0–10)	3.8	2.6	2.4	1.8	< 0.01*	3.9	2.8	2.4	1.8	0.02*	3.5	2.4	2.5	1.7	1.00
COI—Stiffness (0–16)	4.6	4.1	1.8	2.2	0.03*	4.6	3.9	2.1	2.2	0.58	4.0	5.7	1.5	2.5	0.41
COI—Function (0–16)	5.7	5.3	0.9	1.6	< 0.01*	5.0	5.2	1.6	1.8	< 0.01*	4.0	5.4	1.2	2.1	< 0.01*
COI—Gait (0–20)	6.9	5.1	2.2	3.0	< 0.02*	5.7	5.5	3.2	4.4	< 0.05*	4.4	5.4	2.5	3.7	< 0.05*
COI—QOL (0–12)	5.3	3.3	2.8	2.0	1.00	5.1	2.8	2.7	2.1	1.00	4.7	2.6	2.4	1.2	1.00
COI—Overall score (0–64)	22.4	19.1	7.6	8.2	0.13	20.1	15.7	9.6	9.2	0.16	15.7	14.9	7.6	9.2	0.14
LOAD (0–52)	16.4	13.1	6.4	6.5	< 0.01*	13.1	12.4	7.1	6.9	< 0.01*	13.1	12.4	7.6	7.1	< 0.01*

*CBPI* Canine Brief Pain Inventory, *COI* Canine Orthopedic Index, *HVAS* Hudson Visual Analogue Scale, *LOAD* Liverpool Osteoarthritis in Dogs, *PIS* Pain Interference Score, *PSS* Pain Severity Score, *QOL* Quality of Life.

*P* values for the comparison of both groups at each follow-up moment are presented. * Indicates significance.

**Table 2:** Mean values (± standard deviation) of digital thermography, weight-bearing and synovial IL-1 and CRP evaluated throughout the study.

Parameters	Treatment day				8 days					15 days					
	Control		Stanozolol		Control		Stanozolol		*p*	Control		Stanozolol		*p*	
	Mean	SD	Mean	SD	Mean	SD	Mean	SD		Mean	SD	Mean	SD		
**Digital Thermography**															
DV (°, mean ± SD)	24.7	1.9	25.1	1.9	25.2	1.3	23.7	1.9	0.04*	24.4	1.6	24.2	1.4	1.00	
DV max (°, mean ± SD)	26.3	1.9	25.9	1.9	25.8	1.0	25.5	1.9	1.00	26.7	1.6	26.1	2.6	1.00	
Lt (°, mean ± SD)	28.7	2.7	25.8	2.2	31.6	2.1	30.1	2.0	< 0.01*	29.7	2.9	29.4	2.4	< 0.01*	
Lt max (°, mean ± SD)	31.9	3.1	27.6	2.1	34.9	1.0	34.7	1.2	1.00	34.9	0.8	34.8	1.1	1.00	
**Synovial fluid**															
IL-1 (pg/mL, mean ± SD)	170.9	120.4	155.0	145.5	72.3	42.4	92.8	81.9	< 0.01*	–	–	–	–	–	
CRP (mg/mL, mean ± SD)	0.4	1.0	0.3	0.3	0.3	1.2	0.2	0.3	0.35	–	–	–	–	–	
**Weight-bearing**															
Symmetry Index (mean ± SD)	24.7	20.3	24.1	13.9	18.7	17.1	21.6	16.4	1.00	23.9	16.3	24.7	18.3	1.00	
Deviation (mean ± SD)	2.8	3.6	4.25	3.5	2.78	1.987	2.65	1.8	0.71	2.94	2.127	2.31	1.9	< 0.05*	

Parameters	30 days					90 days					180 days				
	Control		Stanozolol		*p*	Control		Stanozolol		*p*	Control		Stanozolol		*p*
	mean	SD	mean	SD		mean	SD	mean	SD		mean	SD	mean	SD	
**Digital Thermography**															
DV (°, mean ± SD)	25.3	1.5	25.2	2.9	1.00	26.1	1.2	25.6	1.1	1.00	25.6	1.4	25.8	1.5	1.00
DV max (°, mean ± SD)	25.2	2.1	26.7	2.8	1.00	27.4	1.4	26.9	1.3	0.04*	26.9	1.4	25.9	1.5	0.02*
Lt (°, mean ± SD)	29.8	2.2	29.9	2.2	< 0.01*	28.4	1.8	28.7	1.9	< 0.01*	27.3	1.8	28.3	2.1	< 0.01*
Lt max (°, mean ± SD)	33.9	1.2	34.5	0.9	< 0.01*	30.5	1.9	31.1	2.2	< 0.01*	29.7	1.9	30.1	2.3	< 0.01*
**Synovial fluid**															
IL-1 (pg/mL, mean ± SD)	122.9	108.9	122.6	96.4	0.58	159.6	59.1	139.8	57.2	1.00	184.2	68.5	165.5	64.2	1.00
CRP (mg/mL, mean ± SD)	0.48	0.9	0.7	2.0	1.00	0.4	0.8	0.3	0.7	1.00	0.0	0.0	0.1	0.4	1.00
**Weight-bearing**															
Symmetry Index (mean ± SD)	18.9	12.2	5.6	7.2	< 0.01*	27.4	12.1	11.0	6.9	< 0.01*	27.0	27.9	6.9	7.3	0.01*
Deviation (mean ± SD)	2.5	1.917	1.31	1.2	0.03*	2.72	2.27	1.85	2.8	0.7	2.61	2.973	2.3	3.2	< 0.01*

*CRP* C-reactive protein, *DV* dorsoventral view, *IL-1* Interleukin 1, *LT* lateral view.

*P* values for the comparison of both groups at each follow-up moment are presented. *Indicates significance.

**Figure 2:**
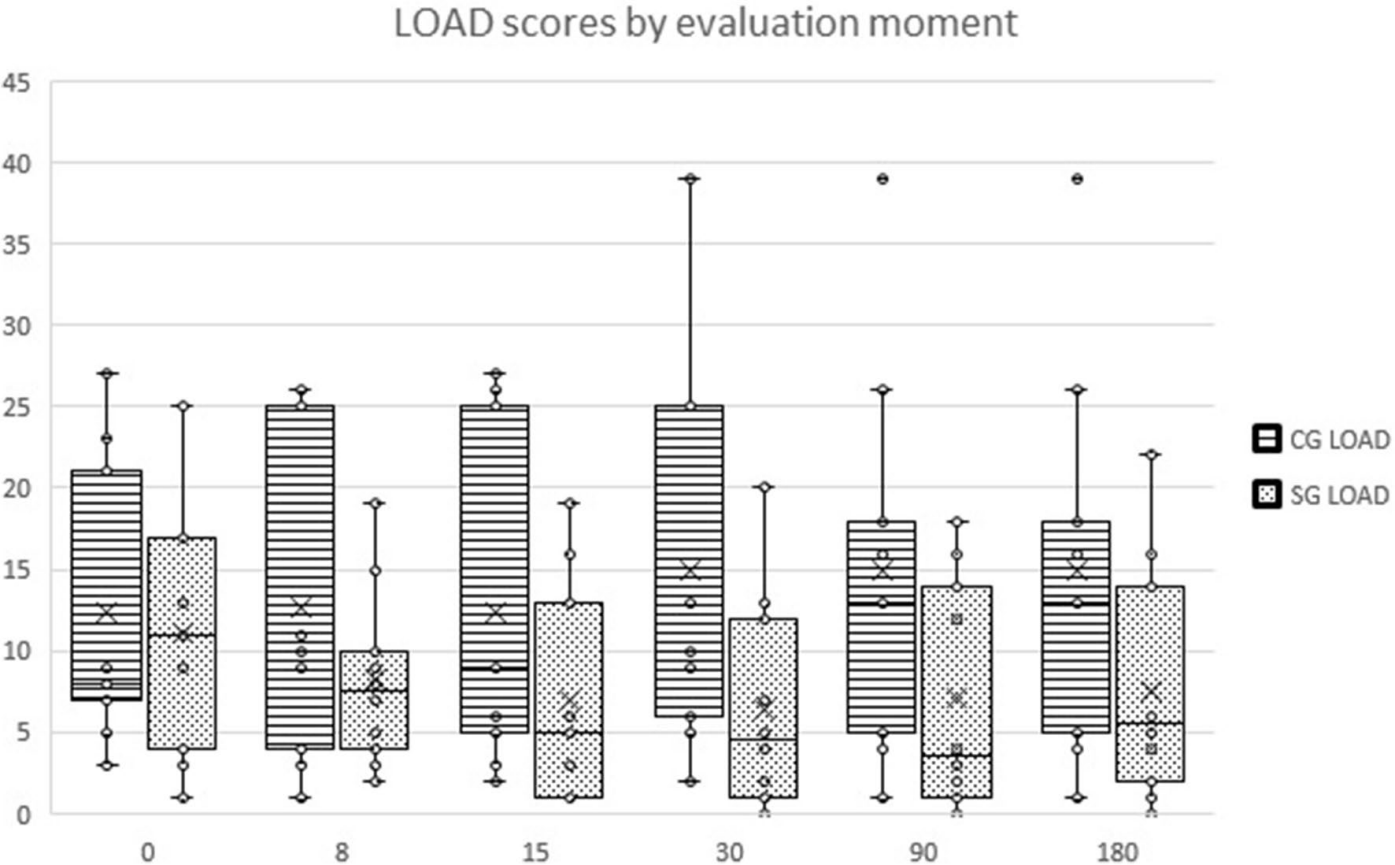
Overall evolution of Liverpool Osteoarthritis in Dogs (LOAD) in the control group and treatment group. Box plots represent the median, 25th and 75th percentiles, and whiskers represent 10th and 90th percentiles.

Significant differences were observed with the different CMI considered, including pain severity score (PSS) (F(3.8, 124.1) = 2.6, *p* = 0.044), pain interference score (PIS) (F(3.7, 117.6) = 3.9, *p* = 0.007), Liverpool Osteoarthritis in Dogs (LOAD) (F(2.5, 81.3) = 3.3, *p* = 0.03), Function (F(2.9, 93.9) = 2.8, *p* = 0.048) and Gait (F(5, 160) = 2.6, *p* = 0.026). Evolution of LOAD is presented in Fig. 2.

The frequency of different radiographic findings at the initial and final evaluations is presented in Table 3. The time to return to baseline values for SI and clinical metrology instruments (CMIs), calculated with Kaplan–Meier estimators, is shown in Table 4.

**Table 3:** Frequency of radiographic findings within the Control and Treatment Groups, at the initial and final evaluations.

Radiographic finding	T0				180d				
	Control		Stanozolol		Control		Stanozolol		*p*
	Absolut	%	Absolut	%	Absolut	%	Absolut	%	
Irregular wear on the femoral head. making it misshapen and with a loss of its rounded appearance	17	85	20	100	20	100	18	90	1.00
Flattened or shallow acetabulum. with irregular outline	11	55	10	50	20	100	16	80	< 0.05*
Caudolateral curvilinear osteophyte (CCO)	5	25	5	25	20	100	15	75	< 0.05*
New bone formation on the acetabulum and on femoral head and neck	20	100	16	80	20	100	18	90	0.16
The angle formed at the cranial effective acetabular rim is worn away	18	90	15	75	20	100	18	90	0.32
Subchondral bone sclerosis along the cranial acetabular edge	19	95	20	100	20	100	18	90	1.00
Circumferential femoral head osteophyte (CFHO)	3	15	9	45	20	100	10	50	0.71

*Indicates significance.

**Table 4:** Time to return to baseline values for SI and CMIs, calculated with Kaplan–Meier estimators and compared with the Breslow test.

Parameters	Breslow test	Treatment			
		Control		Stanozolol	
		Mean ± SD	95% CI	Mean ± SD	95% CI
Simmetry Index	0.022*	47.0 ± 11.8	23.8 ± 70.2	94.2 ± 15.9	62.9 ± 125.4
HVAS	0.000*	48.7 ± 12.4	25.4 ± 73.9	129.8 ± 13.4	103.5 ± 156.1
PSS	0.089	63.2 ± 17.2	29.6 ± 96.8	94.6 ± 16.4	62.5 ± 126.7
PIS	0.000*	8.4 ± 0.4	7.7 ± 9.0	109.6 ± 17.3	75.8 ± 143.2
LOAD	0.000*	40.7 ± 10.6	19.9 ± 61.4	123.8 ± 14.2	95.9 ± 151.6
Stiffness	0.019*	64.7 ± 16.9	31.4 ± 97.9	111.2 ± 15.9	80.6 ± 142.9
Function	0.003*	65.4 ± 13.4	39.2 ± 91.6	124.5 ± 15.4	94.2 ± 154.8
Gait	0.028*	52.7 ± 14.6	23.9 ± 81.4	103.6 ± 15.7	72.8 ± 134.4
QOL	0.656	60.9 ± 15.0	31.4 ± 90.4	66.2 ± 17.5	31.8 ± 100.6
COI	0.122	52.7 ± 13.4	26.5 ± 78.9	78.1 ± 14.0	50.6 ± 105.6

*Indicates significance.

## Discussion

This study describes the effect of a single intra-articular injection of stanozolol, showing that stanozolol had a significant impact on OA joints, improving weight distribution, pain, and function scores compared to the control group. The effect of stanozolol has been studies in different animal models. In horses with naturally occurring OA, a positive response to treatment has been described in 82.5% of cases^22^. The positive effect of stanozolol in this naturally occurring canine model was observed from 15–30 days up to 180 days after treatment, when considering the functional assessment based on weight distribution. Interestingly, this effect was observed even with a single administration, while in the remaining animal models, multiple administrations were carried out^21–23^. This effect is observable in the Kaplan Maier test results for SI, with results of the stanozolol group taking significantly longer to return to baseline values. SI is commonly used to assess lameness, but their calculation with pressure-sensitive walkways has some limitations in OA patients^29^. While it is still unknown if the same limitations apply to the static evaluation of weight-bearing, we looked at different weight-distribution compensation mechanisms by calculating SI and deviation values. It was reasonable to expected improvements in SG only after a relatively large period after the intra-articular administration since stanozolol acts by inducing transforming growth factor 1β synthesis. A further possible stanozolol mechanism of action may be related to its induction in aromatase expression^30^. It has been demonstrated that the human articular cartilage expresses aromatase and that reduced expression of aromatase could facilitate the development of OA^31,32^. Aromatase inhibitor therapy in humans to address other medical conditions might be associated with common musculoskeletal symptoms and with substantial functional disability^33^.

Pain is a hallmark of OA. Data from canine studies may translate to humans^34–36^. Results show that a single intra-articular stanozolol administration significantly improved pain and function scores compared with the control group, raging until the 90-day evaluation moment and, in some cases, until the last evaluation moment. For most of the considered scores, a significant difference was also observed with the Kaplan Meier test. Through the same period, control group scores worsened, as would be expected as the disease progresses. It is interesting to note that some patients in the control group still recorded better scores in follow-up evaluations. This may be due to OA's natural course, with patients sometimes showing spontaneous improvements through time, only to see symptoms reappear in the future. An additional possibility is based on the fact that placebo saline injections can produce an effect, reflected in functional improvements, described to last up to 6-month ^37^. Even though this is possible and may be reflected in some patients' scores, the control group as a whole showed the expected progression of the disease. Additional clinical improvements were observed in the stanozolol group, with improved range of motion during joint extension. A consistent finding with the thermographic evaluation was that higher values were registered in the control group throughout the study, particularly in the last evaluation moments. Digital thermography can assess inflammatory pain and identify osteoarthritic patients^38,39^. Our results seem to support this finding, with higher temperature values determined with this technique corresponding to patients with worse functional evaluation and clinical signs. During digital thermography of dogs, the coat's type and color must be taken into account^40,41^. All of the breeds represented in this study had short hair, some had a double coat, and breeds had similar distribution between groups.

IL-1 is commonly pointed out as a major proinflammatory cytokine responsible for the catabolism in OA in several species, dogs, horses, and humans included^1,42,43^. Therapeutic approaches targeting IL-1 have been developed and shown a positive effect in animal models^44^. The evaluation of synovial fluid can add important information regarding disease burden and progression^45,46^. A previous report has described an improvement in synovial fluid characteristics of animals treated with intra-articular stanozolol^22^. We only observed significant changes at eight days, with both groups showing a reduction from the values recorded at the initial evaluation, but the stanozolol group had higher values. Visual inspection of patients' synovial fluid in the control group at the 8-day evaluation point showed an easily noticeable increased turbidity. The amount of turbidity grossly relates to the amount of inflammation^47^. The stanozolol administration may cause a transient increase in joint inflammation, which may also account for functional improvements, measured with weight distribution, were only observed after this period. Also, since stanozolol acts by inducing transforming growth factor 1β synthesis and reducing nitric oxide, it may not significantly impact IL-1 levels. It is also important to keep in mind that exercise influences inflammatory arthropathies parameters, and increase joint loading adds to secondary inflammation in OA joints^48,49^. As these animals were working dogs, physical activity may also play a role in this finding. The injection of 0.9% NaCl, used in as the control, added to the removal of synovial fluid for analysis, thus removing pro-inflammatory cytokines, may have had a similar effect to that of a joint lavage, and therefore account for the lower IL-1 levels observed in the control group at 8 days.

Radiographic evaluation is still the staple of OA monitoring, with CCO and CFHO representing initial radiographic signs that predict the development of OA clinical signs^50–53^. There is a low relationship between radiographic changes, clinical signs, and limb function^54^. As expected, radiographic signs in the control group progress throughout the follow-up period, representing the natural evolution of OA. In the stanozolol group, the majority of considered radiographic signs did not progress, and some improved. This effect has been described in an ovine surgical induce model, with stanozolol reducing subchondral bone reaction and promoting articular cartilage regeneration^21^. Although the effect has been previously described, future studies have confirmed these changes as no histological samples were collected in this study.

Studies regarding the use of stanozolol in human OA are not performed due to its potential anabolic effects^55,56^. The dose demonstrated to produce anabolic effects is 10 mg twice a week, given through intramuscular administration^57^. In this model, we used the described 0.3 mg/kg dose for intra-articular use in dogs^26,27^. Even if the administered dose may have approached the 10 mg level in some patients, a single administration was used, thus not exceeding the dose needed to produce the anabolic effect. In a study aimed to determine the best intra-articular dose of stanozolol in horses, multiple administrations at the highest dose tested (5 mg) also did not produce any side effects^23^. It is known that after intra-articular administration of stanozolol, it passes rapidly from the joint space to systemic circulation, with maximal plasma concentration registered at 6 h post-administration. It is then eliminated rapidly and detected in plasm for no more than 36 h post local administration^58^. In an ovine model, no weight gain was attributed to the anabolic effect of stanozolol^21^. We also did not recorded significant increases in body weight, which could be attributed to stanozolol. In mice treated with a long-term, high-dose stanozolol regime did not produce significant changes in activity patterns and aggressiveness^18^. No event of aggressiveness or personality changes were reported in treated animals.

Side-effects of intra-articular stanozolol have been previously reported in horses. They include a transient post-injection swelling in the treated joint, which disappeared after a few days without intervention^22^. Similarly, we observed increased lameness in four cases, which spontaneously resolved within a few days. During the follow-up period, no additional medication was administered. The study presents some limitations, namely the fact that the majority of animals had mild OA. It would be of interest to include a larger proportion of animals representing the remaining hip grades. Altough we enroled in the study a similar number of animals to that of similar reports, including a formal sample size calculation and a larger number of patients also is of interest. It is also important to determine the biological significance and clinical relevance of the changes observed. This assessment was made with the Kaplan–Meier test, but the determination of what constitutes a meaningful improvement has not been yet made for some of the evaluations performed. For that reason, we evaluated how long did it take for the assessment to return or drop below the value of the initial presentation, as it was the point which motivated the need for medical assistance. We did not colletect joint histological samples, as this was clinical treatment experiment study. For that reason, the effect of stanozolol on actual disease progression could not be determined on this animal model, and only radiographic progression was evaluated. Further studies should also consider this drug's intra-articular effects, including cytotoxicity, different dose evaluations, and administration frequencies, effect on different parameters as TGF-β synovial levels, similar to what is described in other animal models.

## Conclusions

We described the effect of a single intra-articular administration of stanozolol in a naturally occurring canine model, with a long follow-up period. The use of stanozolol was safe and produced significant improvements in weight-bearing, pain score, and clinical evaluations.

## Methods

This project’s protocol was approved by the ethical review committee of the Universidade de Évora (Órgão Responsável pelo Bem-estar dos Animais, approval nº GD/32055/2018/P1, September 25th, 2018), and complies with the ARRIVE reporting guidelines. All experiments were performed in accordance with relevant guidelines and regulations. For all animals, written, informed consent was obtained from the Institution responsible for the animals (Guarda Nacional Republicana). The sample was composed by forty (N = 40) joints of twenty active Police working dogs with bilateral hip OA. Beeing a convenience sample, it has similar in size to other reports evaluating OA in canine models^59–61^. The diagnosis was based on history (difficulty rising, stiffness, jumping, and maintaining obedience positions), physical examination (pain during joint mobilization, stiffness, and reduced range of motion), and radiographic findings (Orthopedic Foundation for Animals hip scores of mild, moderate or severe). Additional inclusion criteria comprised bodyweight ≥ 20 kg, age > 2 years, and a period > 6 weeks without receiving any medication or nutritional supplements. All inclusion criteria had to be met for the animal to be included in the study. All animals were submitted to a physical, orthopedic, neurological examination, complete blood count, and serum biochemistry. Cases of suspected or documented orthopaedic, neurological, or concomitant disease were excluded. For this prospective, longitudinal, double-blinded, randomly-controlled study, patients were randomly assigned with the statistical analysis software to a control group (CG, n = 20) or a treatment group (SG, n = 20). In SG, an intra-articular (IA) administration of stanozolol (Estrombol, Laboratório Fundacion) at a 0.3 mg/kg dose was administered, while CG received 2 ml of 0.9%NaCl, given IA. Both joints received the same substance, according to the assigned group.

### Weight-bearing evaluation

Weight distribution and off-loading or limb favouring at stance is a commonly used assessment, as it represents limb use and function, and pain^62^. A weight bearing distribution platform was used to perform the weight distribution evaluation (Companion Stance Analyzer; LiteCure LLC®, Newark, Delaware, United States). Conducted procedures followed the manufacturer's guidelines and included placing the equipment in the centre of a room, calibrating it at the beginning of each day, and zeroing it before each data collection. The evaluation itself was conducted with the animal placing one foot on each quadrant of the platform. The patient's head was kept facing forward. The left–right symmetry index (SI) was calculated with the following formula: SI = [(WB_R_-WB_L_)/((WB_R_ + WB_L_) × 0.5)] × 100^63,64^. WB_R_ is the value of weight-bearing for the right pelvic limb, and WB_L_ is the value of weight-bearing for the left pelvic limb. Negative values were made positive. SIs allows for a standardized comparison of ground reaction forces obtained from different individual limbs, eliminating the need to normalize data between subjects. It is considered a specific, sensitive, suitable and reliable assessment of limb dysfunction^29^. Lower SI indicates that the animals is showing a more even distribution of body weight between limbs. Since normal weight-bearing for the pelvic limb is 20%^65^, we also considered the deviation from this value, calculated by subtracting weight-bearing to 20_._

### Digital thermography evaluation

Inflammation in subcutaneous and deeper tissues are reflected in temperature changes in superficial tissues, that can be assessed with digital thermography^66,67^. For collecting digital thermography images, animals were kept for 30 min in a controlled temperature room, with the temperature set at 21 °C. Patients were then placed in an upright standing position, as symmetrical as possible. A dorsoventral image was obtained, including the last lumbar to the first coccygeal vertebrae area, at a distance of 60 cm^68^. From the same position, a lateral view was also obtained, with the greater trochanter at the centre, at the same distance. All images were taken with FLIR ThermaCAM E25® model (FLIR Systems, Wilsonville, Oregon, United States). Thermograms were analyzed with free software (Tools, FLIR Systems, Inc), using a rainbow color pallet. Mean and maximal temperatures were determined by placing boxes of equal size on the hip joint's anatomical area on both views.

### Clinical evaluation

Thigh girth was determined with a Gullick II measuring tape. The patient in was placed in lateral recumbency, with the affected limb uppermost, and the measurement was made at a distance of 70% thigh length, from the tip of the greater trochanter, with an extended leg^69^. Hip joint range of motion was then determined with a goniometer at extension and flexion with a flexed stifle^70^. Pedometers (Xiaomi wrist pedometer) were used to measure the patient's activity levels. They were worn around the patient's neck, attached to an adjustable lightweight collar^71^, for a week before the first evaluation moment to determine a baseline value and then maintained up to the 30th-day post-treatment. For the 90th and 180th post-treatment days evaluation, the pedometer was placed a week before the evaluation moment. Mean daily counts were considered (total number of steps divided by the number of days considered).

### Radiographic evaluation

Pelvic radiographs are frequently performed in dogs to screen hip OA, and are a significant determination of clinical and experimental outcome^54,72,73^. For the IA administrations and radiographic examination, patients were placed under light sedation through the intravenous administration of a combination of medetomidine (0.01 mg/kg) and buthorphanol (0.1 mg/kg). A ventrodorsal extended legs and frog-leg views were obtained during radiographic examination. In the ventrodorsal view, the presence of several radiographic findings was considered: misshapen femoral head with a loss of its rounded appearance; a flattened or shallow acetabulum, with an irregular outline; CCO; new bone formation on the acetabulum and femoral head and neck; a worn away angle formed at the cranial effective acetabular rim; subchondral bone sclerosis along the cranial acetabular edge and CFHO^53,74–76^. In the frog-leg view, the presence of CCO and CFHO was also recorded.

### Treatment administration, synovial fluid collection, and evaluation

With the patients positioned in lateral recumbency, a small window of 4 × 4 cm area surrounding the greater trochanter was clipped and aseptically prepared. An assistant ensured that the limb was placed the limb in a neutral, parallel to the table position. A 21-gauge 2.5" length needle was introduced just dorsal to the greater trochanter and perpendicular to the table, until the joint was reached^77^. A collected of synovial fluid ensured correct needle placement was obtained by collecting synovial fluid, and the treatment or saline was administered. The syringes containing the substance to be administrated were prepared by a different researcher and covered to hyde the substance's characteristics and keep the treatment administrator blinded to the treatment. A sample of synovial fluid was saved for the determination of interleukin-1β (IL-1β), made DuoSet Ancillary Canine IL-1β Reagent kit (R&D Systems, UK). Plates were read with a FLUOstar OPTIMA (BMG Labtech). C-reactive protein (CRP) concentrations were made using the Fuji Dri-Chem Slides VC-CRP PS (FUJIFILM Europe GmbH), and read with a DRIChem NX500i (FUJIFILM Europe GmbH). Additionally, dogs' trainers completed a copy of HVAS, CBPI, COI, and LOAD after receiving the published instructions for each of them. They were completed in sequence by the same trainer in a quiet room with as much time as needed to answer all items.

After treatment, animals were rested for three days, resuming normal activity over five days. Signs of increased pain, persistent stiffness, and changes in posture exhibited by the dogs, were evaluated by the veterinarian on days 1 and 3 after the IA administration^78,79^. Follow-ups were conducted on days 0 (treatment day), 8, 15, 30, 90, and 180. An outline of all procedures at each moment is presented in Table 5. The same researcher, blinded to the animal's assigned group and identification and moment of evaluation, performed all assessments. For the radiographic and digital thermography evaluation, all personal information was removed before the evaluation. After the study, all patients remained in active Police work.

**Table 5:** Procedures conducted at each moment.

Modality	Evaluation moment					
	0 treatment day	8	15	30	90	180
Stance analysis	X	X	X	X	X	X
Digital Thermography	X	X	X	X	X	X
Pedometer	X	X	X	X	X	X
Goniometry	X	X	X	X	X	X
Thigh girth measurement	X	X	X	X	X	X
Digital radiography	X			X	X	X
Treatment	X					
SF CRP	X	X		X	X	X
SF IL-1	X	X		X	X	X
HVAS	X	X	X	X	X	X
CBPI	X	X	X	X	X	X
COI	X	X	X	X	X	X
LOAD	X	X	X	X	X	X

Days are counted from treatment day.

*CBPI* Canine Brief Pain Inventory, *COI* Canine Orthopedic Index, *CRP* C-Reactive Protein, *HVAS* Hudson Visual Analogue Scale, *IL-1* Interleukin 1, *LOAD* Liverpool Osteoarthritis in Dogs, *SF* Synovial fluid.

### Statistical analysis

Normality was assessed with a Shapiro–Wilk test. Groups' results were compared in each evaluation moment, and each measured parameter was compared with the result observed on treatment day. Results were compared with a Paired samples t-test, Repeated Measures ANOVA, with a Huynh–Feldt correction, or Wilcoxon signed-ranks test to assess the effect of different parameters on the patients' clinical evolution. A Kaplan–Meier test was conducted to evaluate the time to return to baseline values of SI and CMI scores. Results were compared with the Breslow test. As for the CBPI a specific measure of success has been determined as a reduction of ≥ 1 in PSS and ≥ 2 in PIS^80^, the Kaplan–Meier test was used to evaluate the time for the score to drop below this reduction level in these scores. All results were analysed with IBM SPSS Statistics version 20 (IBM Corporation, New York, USA), *p* < 0.05.

## Data Availability

The datasets used and/or analyzed during the current study are available from the corresponding author on reasonable request.
